# Insulin-like growth factor binding protein 5 (IGFBP5) functions as a tumor suppressor in human melanoma cells

**DOI:** 10.18632/oncotarget.4114

**Published:** 2015-05-12

**Authors:** Junyun Wang, Nan Ding, Yongjun Li, Hua Cheng, Dong Wang, Qiong Yang, Youhui Deng, Yaran Yang, Yanming Li, Xiuyan Ruan, Fang Xie, Hua Zhao, Xiangdong Fang

**Affiliations:** ^1^ CAS Key Laboratory of Genome Sciences and Information, Beijing Institute of Genomics, Chinese Academy of Sciences, Beijing, China; ^2^ University of Chinese Academy of Sciences, Beijing, China; ^3^ Institute of Biology, Hebei Academy of Sciences, Shijiazhuang, China; ^4^ Department of Dermatology, General Hospital of People's Liberation Army, Beijing, China

**Keywords:** IGFBP5, malignant melanoma, tumor suppressor, EMT, ERK-MAPK

## Abstract

The insulin-like growth factor binding protein 5 (IGFBP5), which is often dysregulated in human cancers, plays a crucial role in carcinogenesis and cancer development. However, the function and underlying mechanism of IGFBP5 in tumor growth and metastasis has been elusive, particularly in malignant human melanoma. Here, we reported that IGFBP5 acts as an important tumor suppressor in melanoma tumorigenicity and metastasis by a series of experiments including transwell assay, xenograft model, *in vivo* tumor metastasis experiment, and RNA-Seq. Overexpression of IGFBP5 in A375, a typical human melanoma cell line, inhibited cell malignant behaviors significantly, including *in vitro* proliferation, anchorage-independent growth, migration and invasion, as well as *in vivo* tumor growth and pulmonary metastasis. In addition, overexpression of IGFBP5 suppressed epithelial-mesenchymal transition (EMT), and decreased the expression of E-cadherin and the key stem cell markers NANOG, SOX2, OCT4, KLF4, and CD133. Furthermore, IGFBP5 exerts its inhibitory activities by reducing the phosphorylation of IGF1R, ERK1/2, and p38-MAPK kinases and abating the expression of HIF1α and its target genes, VEGF and MMP9. All these findings were confirmed by IGFBP5 knockdown in human melanoma cell line A2058. Taken together, these results shed light on the mechanism of IGFBP5 as a potential tumor-suppressor in melanoma progression, indicating that IGFBP5 might be a novel therapeutic target for human melanoma.

## INTRODUCTION

The insulin-like growth factor binding proteins (IGFBPs) comprise a family of six proteins that function as critical regulators of the bioavailability and mitogenic activities of insulin-like growth factors (IGFs). IGFBP5, the most conserved member of the IGFBPs family, is frequently dysregulated in human cancers and metastatic tissues [1, 2]. IGFBP5 has several functional roles in carcinogenesis and cancer development, which can determine cell survival and regulate cell growth, migration, and invasion in the development of cancer. Many preclinical studies indicate that IGFBP5 can suppress tumor growth and metastasis in various tissues and contexts, but IGFBP5 can also function as an oncogene, promoting metastasis in a context-dependent manner [2-4]. IGFBP5 modulates cell functions by both IGF-dependent mechanisms, which affect IGF 1 receptor (IGF1R) signaling, and IGF-independent mechanisms that do not alter IGF1R signaling [3]. Furthermore, the different domains of IGFBP5 exert distinct effects on the tumorigenicity and metastasis of different human cancers [5, 6].

Despite the increasing number of studies supporting the role of IGFBP5 in tumorigenesis and metastasis in several types of cancers, its function in the progression of cancer is controversial and few studies have provided mechanistic insights for IGFBP5 in human malignant melanoma (MM). MM is one of the most lethal forms of skin cancer, and its incidence has been rising for, at least, the past 30 years [7, 8]. In patients with MM, tumor metastasis is highly aggressive and is the leading cause of mortality. The molecular mechanisms underlying the development and progression of melanoma are still not well understood, demonstrating the need for novel diagnostic markers and therapeutic targets to combat MM. Other IGFBPs family members, such as IGFBP3, have been associated with melanoma progression [9-12]. Thus, it is imperative to determine the roles of IGFBP5 in melanoma.

Our previous study revealed that IGFBP5 exhibited a distinctly different expression pattern in melanoma cells with RNA-sequencing (RNA-Seq) [13]. Hence, in order to define the role of IGFBP5 in MM growth and metastasis, we investigated the effects of IGFBP5 in A375 and A2058 human melanoma cells and its potential as a potent tumor growth inhibitor and anti-metastatic agent both *in vitro* and *in vivo*, using stable overexpression and knockdown cells. Our findings provide new insights into the mechanism by which IGFBP5 suppresses the proliferation and invasion of melanoma cells.

## RESULTS

### IGFBP5 expression is associated with melanoma

**Figure 1:**
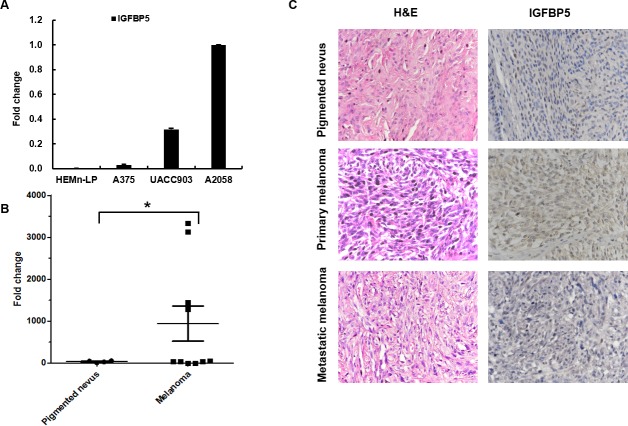
IGFBP5 expression in cell lines and clinical melanoma samples **A.** The relative endogenous expression of *IGFBP5* analyzed for in HEMn-LP and the 3 MM cell lines, A375, UACC903, and A2058 by qRT-PCR. *GAPDH* was used as the internal control. **B.** qRT-PCR analysis of the expression of IGFBP5 in normal pigmented nevus samples (*n* = 5) and melanoma samples (*n* = 10) collected from General Hospital of PLA. Data were shown for the mean ± standard deviation (SD) from three independent experiments. *, *P* < 0.05. **C.** Representative H&E and immunohistochemical (IHC) stains of a normal pigmented nevus, a primary melanoma tissue, and a lymph node metastatic tissue. The mean IHC scores of the melanoma tissues and the pigment nevus tissues were 1.8 and 0.4. *, *P* < 0.05. IGFBP5 staining was intense in the primary tumor tissues and that was weak in the normal pigmented nevus and metastatic tumor tissues. The magnifications of the images were × 400.

A critical question that has been raised is whether the expression of IGFBP5 clinically correlates with the progression of human melanoma. To address this issue, we studied IGFBP5 expression in HEMn-LP normal melanocytes and three human melanoma cell lines (A375, A2058, and UACC903) using quantitative real-time PCR (qRT-PCR). We found that IGFBP5 was highly expressed in A2058 and UACC903 cells, but low expression in HEMn-LP and A375 cells relatively (Figure 1A).

In clinical samples, the expression of IGFBP5 in melanoma samples (*n* = 10) is higher than in normal pigmented nevus samples (*n* = 5) significantly by qRT-PCR analysis (*, *p* < 0.05, Figure 1B). Furthermore, we analyzed the expression of IGFBP5 by hematoxylin-eosin (H&E) and immunohistochemical (IHC) staining in human pigmented nevus samples (*n* = 7), primary human melanoma samples (*n* = 7), and human metastatic melanoma samples (*n* = 8). IHC staining was graded in four categories: IHC 3 +, 2 +, 1 + and 0 -. Our results revealed that the mean IHC score for all the melanoma samples was 1.8 compared to 0.4 for the pigment nevus tissues (*, *p* < 0.05). Figure 1C illustrates the strong staining for IGFBP5 from a primary melanoma sample compared to the weak staining from a metastatic tissue and a normal pigmented nevus sample.

### IGFBP5 inhibits melanoma cell proliferation and suppresses tumor growth *in vivo*

**Figure 2:**
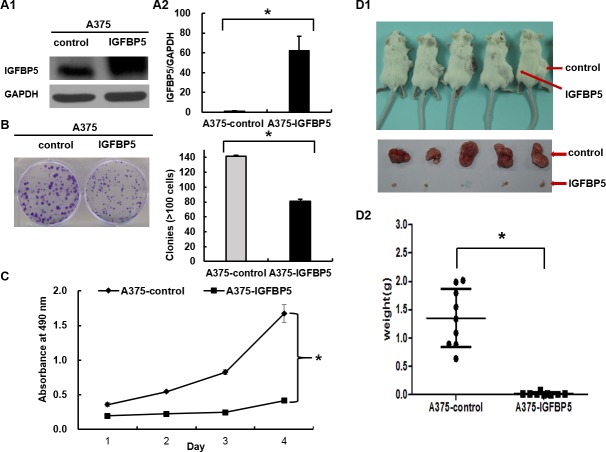
IGFBP5 inhibits melanoma cell proliferation both i*n vitro* and *in vivo* **A.** A375 melanoma cells were transfected with pcDNA3.1-Neo-EGFP-IGFBP5 and empty pcDNA3.1-Neo-EGFP vectors. Western blots (left) and qRT-PCR (right) analyses were used to detect IGFBP5 expression in transfected cells. Colony formation assay **B.**, CCK-8 assay **C.**, and xenograft assay **D.** were used to explore the effects of stable IGFBP5 overexpression on melanoma cell proliferation. Data were shown for the mean ± SD from three independent experiments. *P* values based on two-side Student *t*-test comparing control and IGFBP5 OE. *, *P* < 0.05.

To investigate the role of IGFBP5 in melanoma progression, we stably transfected the human melanoma cell line A375 with IGFBP5, which enabling high expression of IGFBP5, as shown with western blot (WB) and qRT-PCR (Figure 2, panels A1 and A2). Using this stable IGFBP5 overexpression (OE) cell line, we assessed the effects of IGFBP5 on cell proliferation and tumor growth using CCK-8 assay and colony formation assay. Our results demonstrate that elevated expression of IGFBP5 inhibited cell proliferation significantly *in vitro*. (Figure 2, panel B and C).

To confirm the inhibitory effects of IGFBP5 on tumor growth *in vivo* further, A375 vector control and OE cells were implanted subcutaneously into the abdomens of SCID/Beige mice. As a result, all mice developed tumors at their injection sites. Remarkably, IGFBP5 inhibited tumor growth in IGFBP5 OE mice significantly (mean tumor weight: 0.018 ± 0.008 g, *, *p* < 0.05), whereas the tumors of the control group grew far larger (mean tumor weight: 1.73 ± 0.46 g) (Figure 2D).

We further investigated the function of IGFBP5 using stable IGFBP5 knockdown (KD) A2058 cells. The expression of IGFBP5 decreased by 90% compared to the control by WB and qRT-PCR analyses. Consistent with IGFBP5 overexpression results, down-regulation of IGFBP5 promoted cell proliferation and tumor growth significantly (Figure S1). Together, these data substantially demonstrate that IGFBP5 functions as a tumor suppressor for melanoma tumor growth.

### IGFBP5 represses tumor cell migration, invasion, and suppresses pulmonary metastasis *in vivo*

**Figure 3:**
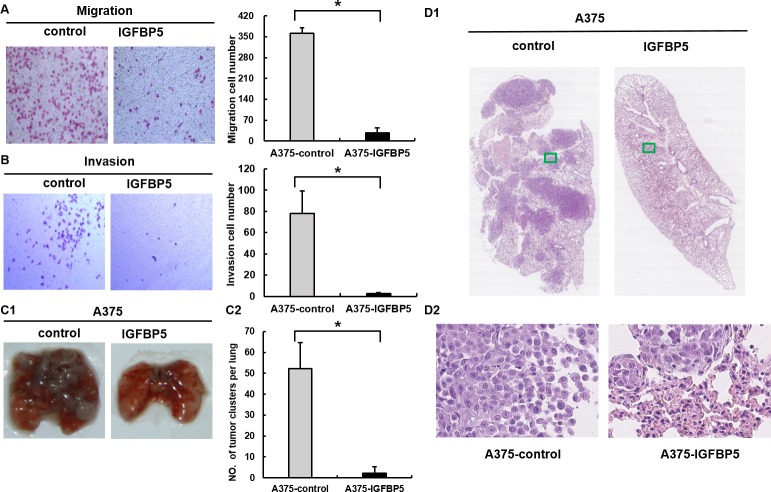
IGFBP5 inhibits cell migration and invasion *in vitro* and suppresses pulmonary metastasis *in vivo* Transwell cell migration assay **A.** and Matrigel cell invasion assay **B.** were conducted between control cells and A375 IGFBP5 OE cells. Representative images of cells stained with H&E (left) and the mean of migrated and invaded cells (right) were shown. Data were shown for the mean ± SD from three independent experiments. *, *P* < 0.05. (C1) Representative images of the lungs harvested from mice injected with vector control cells and A375 IGFBP5 OE cells were shown. (C2) The mean number of metastatic lung clusters from mice control and A375 IGFBP5 OE tumors were plotted, as analyzed by H&E staining. *, *P* < 0.05. (D1 and D2) H&E staining of lung tumor sections and adjacent tissues from control and IGFBP5 OE mice. Representative images of the harvested lungs from injected mice were shown for each treatment. The images in D2 are the magnifications of the two green boxes in D1.

To explore the effects of IGFBP5 on MM progression, we conducted a series of tumor migration and invasion assays *in vitro* with stably transfected A375 OE cells and *in vivo* in xenograft mice. Up-regulation of IGFBP5 markedly inhibited cell migration through a permeable filter (92% suppression) and invasion through a Matrigel matrix (96% suppression) compared to controls (*, *p* < 0.05, Figure 3, panels A and B). Conversely, down-regulation of IGFBP5 promoted cell migration and invasion significantly (*, *p* < 0.05, Figure S2, panels A and B). Subsequently, we performed pulmonary metastasis assays in SCID/Beige mice. The pulmonary metastatic clusters, which presented in the mice with OE cells (2.2 ± 3.3 clusters per lung, *, *p* < 0.05), were significantly fewer than those in the control group (52.3 ± 12.3 clusters per lung), as shown by H&E staining. Notably, overexpression of IGFBP5 rarely formed secondary metastases in the lungs of mice, whereas control mice were found to have extensive and severe metastatic deposits in both lungs (Figure 3, panel C and D).

We next examined the effects of IGFBP5 KD on A2058 cell migration and metastasis and observed that down-regulation of IGFBP5 promoted the formation of micrometastases in the IGFBP5 KD mice. Pulmonary metastases were easily detected when endogenous expression of IGFBP5 was knocked down in A2058 cells (95.8 ± 38.8 clusters per lung, *, *p* < 0.05.), whereas fewer and smaller metastases were observed in scramble control mice (27.3 ± 20.0 clusters per lung) (Figure S2, panels C1 and C2). Representative H&E staining of lung tumor sections and adjacent tissues from IGFBP5 knockdown mice and control mice was shown (Figure S2, panels D1 and D2). Collectively, these observations indicate that IGFBP5 negatively regulated the metastatic properties of melanoma cancer cells both *in vitro* and *in vivo*.

### IGFBP5 suppresses EMT and stem cell features of tumor cells

**Figure 4:**
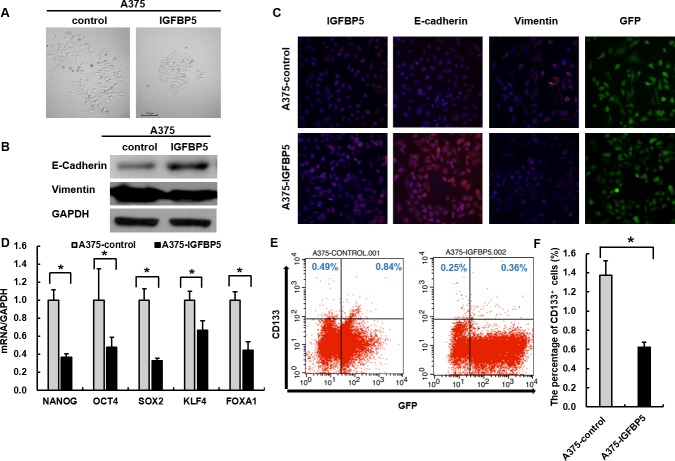
IGFBP5 suppresses EMT and stem cell features of tumor cells **A.** Representative images illustrating the characteristic morphology of A375 control and IGFBP5 OE cell clones were shown. Magnification, × 200. The expression of EMT markers was analyzed by western blots **B.** and immunofluorescence **C.** in A375 control and IGFBP5 OE cells. Nuclei were stained with DAPI. Magnification, × 200. **D.** Assessment of relative expression levels of representative stem cell markers by qRT-PCR. Data were shown for the mean ± SD form three independent experiments. *P* values based on two-side Student *t*-test comparing vector control cells and A375 IGFBP5 OE tumor cells. *, *P* < 0.05. **E.** FACS analysis of stem cell marker CD133 in IGFBP5 OE cells. **F.** Graph demonstrates the mean ± SD for the percent of CD133^+^ cells from three independent experiments. *, *P* < 0.05.

Given that IGFBP5 inhibited cancer metastasis and characteristic morphological changes were observed in A375 and A2058 (Figure 4A and Figure S3A), we investigated whether IGFBP5 plays a role in regulating EMT, a critical event in tumor invasion and progression. Accordingly, we tested EMT-associated markers with WB and immunofluorescence and found that A2058 cells transfected with IGFBP5-targeting shRNA exhibited prominent mesenchymal-like phenotypes, including a fibroblast-like morphology. We also observed an increase in the expression of the mesenchymal marker vimentin (VIM) and a decrease of the epithelial marker E-cadherin (ECAD) (Figure S3, panels A - C). However, overexpression of IGFBP5 in A375 cells led to a cobblestone morphology in monolayer cultures with tight cell-cell contacts, characteristic of normal epithelial cells (Figure 4A), indicating that IGFBP5 most likely reversed tumor cell EMT. This morphological change accompanied by an increase in the expression of ECAD and a decrease in VIM. These results were also confirmed by WB and immunofluorescence assays (Figure 4B and 4C).

To determine whether IGFBP5-inhibited EMTs affect the generation of cancer stem cell (CSC)-like cells, we performed qRT-PCR to analyze the master stem cell transcription factors (TFs) in OE cells. We found that overexpression of IGFBP5 decreased expression of the stem cell TFs: NANOG, SOX2, KLF4, and OCT4, at the transcriptional level (Figure 4D), and the expression of these stem cell TFs were increased in IGFBP5 KD A2058 cells (Figure S3D). Furthermore, we tested the expression of the stem cell marker, CD133, in melanoma clones. IGFBP5-silenced A2058 cells acquired a higher CD133 expression phenotype than control cells (Figure S3, panels E and F). By contrast, A375 OE cells showed a reduced CD133 expression phenotype compared to GFP control cells (Figure 4, panels E and F).

**Figure 5:**
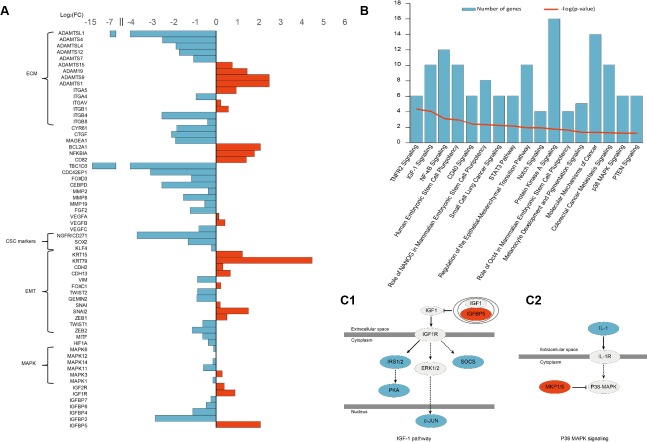
RNA-Seq Analysis of IGFBP5 overexpression in A375 cells **A.** Genes involved in tumor progression differentially expressed between IGFBP5 OE and empty vector control A375 cells. **B.** The most altered pathways identified with the Ingenuity Pathway Analysis (IPA) are listed, including IGF1 signaling and p38-MAPK signaling pathways. (C1 and C2) The IGF1 signaling and p38-MAPK signaling pathways were inhibited in IGFBP5 OE cells. Red color indicates up-regulation and blue color indicates down-regulation in IGFBP5 OE cells.

We sequenced the RNA transcriptome of A375 IGFBP5 OE cells using RNA-Seq methodology and found that the changes in the expression of the EMT markers were consistent with the variations detected with both the WB and immunofluorescence assays. The melanoma CSC markers SOX2, KLF4, and CD271 exhibited reduced expression profiles in the A375 IGFBP5 OE cells compared to empty vector control cells (Figure 5A). Intriguingly, genes from the “Regulation of the EMT Pathway” and three other pathways related to stem cell pluripotency (“Human Embryonic Stem Cell Pluripotency”, ”Role of NANOG in Mammalian Embryonic Stem Cell Pluripotency”, and “Role of OCT4 in Mammalian Embryonic Stem Cell Pluripotency”), were categorized among those most altered with the Ingenuity Pathway Analysis (IPA) software (Figure 5B). Taken together, these results suggest that IGFBP5 inhibited both the EMT procession and stem cell properties of melanoma cells.

### IGFBP5 affects melanoma growth and metastasis through inhibition of the extracellular signal-regulated kinase (ERK) and p38-MAPK signaling pathways

**Figure 6:**
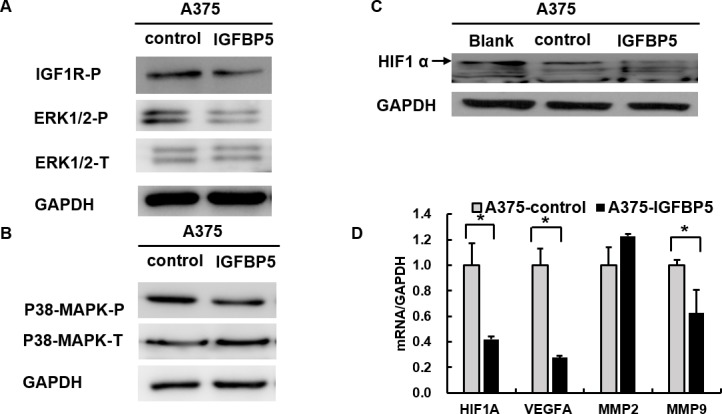
IGFBP5 inhibits HIF1α expression through the MAPK-ERK signaling pathway **A.** and **B.** Western blots using specific antibodies for the phosphorylation state of IGF1R, ERK1/2, and p38-MAPK from A375 IGFBP5 OE and control cells. Phosphorylation of IGF1R, ERK1/2, and p38-MAPK was decreased in A375 cells transfected with IGFBP5. GAPDH was used as a loading control. **C.** Overexpression of IGFBP5 inhibited HIF1α expression visualized by western blots of A375 cells. The arrow points to the band of HIF1α. **D.** Assessment of the reduced gene expression levels of *VEGFA* and *MMP9*, downstream genes regulated by HIF1α, in A375 IGFBP5 OE compared to control cells by qRT-PCR analysis. Data were shown for the mean ± SD form three independent experiments. *P* values based on two-side Student *t*-test comparing A375 IGFBP5 OE tumor cells and vector control cells. *, *P* < 0.05.

It is well established that human MM is driven by the activation of ERK-MAPK signaling, and most frequently through mutations in the BRAF or NRAS oncogenes. To test whether IGFBP5 suppressed melanoma growth and metastasis through inhibition of this signaling pathway, we examined the phosphorylation of ERK and p38-MAPK and found that their phosphorylation levels decreased in A375 IGFBP5 OE cells and increased in A2058 IGFBP5 KD cells (Figures 6, panels A and B, and Figures S4, panels A and B). Furthermore, we found IGFBP5 down-regulated the expression of the hypoxia-inducible factor 1 α (HIF1α) in A375 IGFBP5 OE cells, and the genes targeted by HIF1α, VEGFA and MMP9, were also dramatically reduced. Similarly, we also observed that HIF1α increased in IGFBP5 KD cells, and VEGFA and MMP9 were correspondingly elevated 18.3-fold and 7.5-fold, respectively. These results imply that IGFBP5 may mediate HIF1α expression to inhibit tumor growth and metastasis (Figures 6, panels C and D, and Figures S4, panels C and D).

To determine the molecular mechanisms of IGFBP5 functions in MM, we performed RNA-Seq analysis of A375 IGFBP5 OE cells using the Ion Proton™ system. We analyzed the differences in gene expression and gene function with IPA software, and the results confirmed our experimental findings (Figure 5, panels A and B). The IGF1 and p38-MAPK signaling pathways were inhibited (Figure 5C), and the expression level of the EMT-related TFs, TWIST1 and ZEB2, is inversely related to IGFBP5 level. Specifically, the mRNA level of IGFBP5 was low, whereas that of TWIST1 and ZEB2 were high. Moreover, the expression of the TBC1D3 oncogene, melanocyte-specific transcription factor/microphthalmia associated transcription factor (MITF), HIF1α regulated gene CEBPD, the Rho GTPase member CDC42, growth factors CTGF and FGF2, and β4 integrin gene ITGB4 all decreased dramatically. These genes regulate multiple cellular activities, including cytoskeleton reorganization, Rho GTPase effector functions, cell-matrix or cell-cell adhesion, and transduction signals that regulate gene expression, cell growth, cell invasion, and metastasis (Figure 5A and Table S1).

## DISCUSSION

Although IGFBP5 has been associated with various types of cancers, acting in oncogenic or tumor-suppressive roles, such as breast cancer [4, 6, 14-16], osteosarcoma [5, 17, 18], head and neck squamous cell carcinoma [19], neuroblastoma [20], or prostate cancer [21], little is known about the role of IGFBP5 in human MM. In this study, we identify IGFBP5 as a novel inhibiting factor of tumor growth and metastasis in melanoma.

By modulating the tissue distribution of IGFs and their access to cell receptors, IGFBPs limit the bioavailability of IGFs, which are implicated in tumorigenesis [22]. The mechanism of IGFBP5 as a regulator of the oncogenic receptor ligands IGF-I and -II is well understood [23, 24], but there are relatively few studies demonstrating the involvement of IGFBP5 in the signaling pathways that regulate tumor growth and metastasis. Previous studies report that IGFBP5 influences pancreatic cancer cell growth and survival via the MAPK or PI3K pathway, and enhances prostate cancer growth through activation of the PI3K pathway [25, 26]. It is widely accepted that MM is driven by the activation of MEK-ERK signaling pathway, typically through mutations in the BRAF or NRAS oncogenes, as well as upstream membrane receptors (e.g. EGFR and IGF1R) [27-32]. p38-MAPKs also play a vital role in the progression of melanoma [33, 34]. Therefore, we propose that IGFBP5 suppresses tumor growth and metastasis, including EMT, through the MEK-ERK and p38-MAPK signaling pathways. We found that the phosphorylation of ERK1/2, p38-MAPK, and IGF1R was attenuated in IGFBP5 OE cells, indicating that IGFBP5 inhibited cell proliferation and metastasis through the IGF1R-dependent pathway. Conversely, the phosphorylation of these kinases was enhanced in IGFBP5 KD cells. Furthermore, RNA-Seq pathway analysis revealed that the IGF1 and p38-MAPK signaling were inhibited in IGFBP5 OE cells.

The expression of HIF1α, the master regulator of tumorigenesis, tumor metastasis, and development, increased with the activation of ERK1/2-MAPK pathway in human melanoma [35-38]. We observed that the expression of HIF1α was inhibited in melanoma cells overexpressing IGFBP5, which is consistent with the inhibition of ERK1/2 and MAPK activities. Accordingly, the expression of the genes targeted by HIF1α, VEGF and MMPs, were downregulated in IGFBP5 OE cells. Hypoxia levels were reduced in tumor cells, resulting in decreased levels of metastasis-promoting genes (MMPs, VEGFs, FGF2, and CEBPD) thus impairing metastasis [39]. In addition, HIF1α can induce the expression of IGFBPs family members, including IGFBP2 [40], IGFBP3 [41], and IGFBP6 [42], whereas HIF1α expression is regulated by IGF1 signaling trigged by IGFBP1 and IGFBP2 [40-43]. In our study, we first found that up-regulation of IGFBP5 decreased HIF1α expression and the other IGFBPs family members, IGFBP2, IGFBP4, IGFBP6, and IGFBP7 to some extent in melanoma cells. However, the relationship between HIF1α and IGFBP5 in human melanoma cells remains to be verified.

Our data point to a negative correlation between IGFBP5 expression and EMT phenotypes. EMT is characterized by the loss of cell polarity, down-regulation of epithelial proteins most prominently ECAD, and up-regulation of the mesenchymal protein, VIM, which has more recently been implicated in promoting carcinoma invasion and metastasis [44, 45]. Moreover, an increasing number of studies show that the progression of EMT can generate tumor cells with properties of CSCs [46-50]. Vijayan et al. revealed that IGFBP5 enhances epithelial cell adhesion and protects epithelial cells from TGFβ1-induced mesenchymal invasion in NMuMG cells [51]. In our study, we found that overexpression of IGFBP5 inhibits EMT and decreases the key stem cell markers NANOG, SOX2, OCT4, KLF4, whereas the knockdown of IGFBP5 leads to a repression of the epithelial phenotype, inducing a mesenchymal-like phenotype, and increasing migratory and invasive behaviors and CSC-like properties. Additionally, we purport that the IGFBP5 might suppress expression of ZEB2 and TWIST1 at the transcriptional level from the results of our RNA-Seq analysis. ZEB2 is a member of the zinc finger homeodomain enhancer-binding protein (ZEB) family, and it is both necessary and sufficient to repress ECAD transcription and trigger the EMT process in melanoma [52, 53]. For this reason, we believe that IGFBP5 suppresses EMT, at least partially, by inhibiting TWIST1 and ZEB2. We found that the CSC markers SOX2, KLF4, and CD271 (also known as NGFR) decrease dramatically in IGFBP5 OE cells in our RNA-Seq data, and stem cell pluripotency-related pathways were inhibited in our IPA analysis.

Analysis of our RNA-Seq data permitted an investigation of the causes and molecular mechanisms of IGFBP5 functions in IGFBP5 OE cells. Foremost, we found that many top down-regulated genes were related to cell-cell adhesion, cell-ECM adhesion, ECM proteins, and growth factors and receptors, which all promoted tumor growth and progression. Next, we found that overexpression of IGFBP5 not only resulted in a widespread oscillatory-like pattern of changes in gene expression, but also disrupted both canonical and non-canonical signaling pathways, including the IGF-1 signaling, NF-κB signaling, Notch signaling, p38-MAPK signaling, EMT, stem cell pluripotency, and PTEN signaling pathways. This suggests that IGFBP5 may act as a tumor suppressor disturbing a wide array of signaling pathways. In the future, we plan to utilize proteomics techniques to further explore the changes of protein expression profiles and hope to find new factors that contribute to the inhibition of melanoma progression induced by the overexpression of IGFBP5.

**Figure 7:**
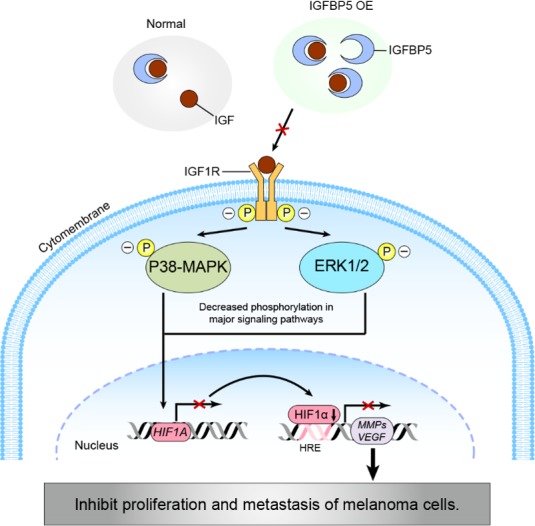
A schematic diagram modeling a potential pathway for the IGFBP5-IGF1R-MAPK-HIF1α signaling inhibition of melanoma tumor cell growth and metastasis IGFBP5 represses the proliferative and metastatic capabilities of cancer cells by (1) inhibiting ERK1/2 and p38-MAPK activities through an IGF1R-dependent signaling pathway and (2) down-regulating the expression of HIF1α through the transcriptional inhibition of HIF1A gene expression, resulting in a decrease in VEGF and MMPs expression.

In summary, we have identified IGFBP5 as a novel suppressor of the pathogenesis and metastasis of malignant melanoma by expression-manipulating experiments (Figure 7). We have demonstrated that IGFBP5 suppresses melanoma cell growth and metastasis through inhibition of the ERK1/2 and P38-MAPK pathways. Because IGFBP5 appears to exert a specific inhibitory effect on melanoma growth and metastasis, it may qualify as a useful therapeutic target against melanoma and, perhaps, other cancers.

## MATERIALS AND METHODS

### Cell culture and tissue samples

Normal human melanocytes, neonatal, lightly pigmented donor (HEMn-LP), and the malignant human melanoma cells, A375 and A2058, were purchased from ATCC (Manassas, VA, USA). The human melanoma cell line, UACC903, was a gift from Dr. Yongliang Zhao (Beijing Institute of Genomics, CAS). All melanoma cell lines were maintained in Dulbecco's modified Eagle's medium (DMEM, Gibco, Grand Island, NY, USA) supplemented with 10% (v/v) fetal bovine serum (FBS, AusGeneX, Molendinar, Qld, Australia) and penicillin (100 U/mL)-streptomycin (0.1 mg/mL) (Invitrogen, Carlsbad, CA, USA). All cells were cultured in a 37°C humidified, 5% CO_2_ atmosphere incubator (Thermo Fisher Scientific, Waltham, MA, USA).

The acquisition of tissue samples was approved by the Chinese PLA General Hospital. We used primary melanoma samples, metastatic melanoma samples, and normal pigmented nevus samples to test the endogenous expression of IGFBP5. Informed consent was given by all patients examined. All human samples were collected in accordance with the “Declaration of Helsinki” (as revised in Edinburgh 2000).

### RNA isolation and quantitative real-time PCR

Total RNA was extracted from cells and tissues using TRIzol^®^ Reagent (Life Technologies, Carlsbad, CA, USA) according to the manufacturer's instructions, and equal mass amounts (1 μg) of RNA was reverse transcribed and the cDNA was used in duplicate real-time PCRs using Maxima SYBR green/ROX qPCR master mix (Fermentas, Amherst NY, USA). Relative gene expression levels were calculated using CFX Manager Software. GAPDH was used as an internal control. The primer sequences for all qRT-PCR experiments were provided in Table S2.

### Cloning and plasmid construction

Human IGFBP5 cDNA, which codes for the 272 amino acids of the IGFBP5 protein, was amplified by PCR from normal human blood with Pfu DNA Polymerase (M7741, Promega, Madison, WI, USA) and cloned into pcDNA3.1-Neo-EGFP plasmid (Invitrogen, Carlsbad, CA, USA). The cloning primers for IGFBP5 were: 5′-CCCCTCGAGATGGTGTTGCTCACCGCGGT-3′ (forward) and 5′- CCCGCGGCCGCTCACTCAACGTTGCTGCTGT-3′ (reverse).

A375 cells were stably transduced with the IGFBP5 expression vector (pcDNA3-Neo-EGFP-IGRBP5), using Lipofectamine^®^ 2000 according to the manufacturer's protocol. The short-hairpin RNA (shRNA) targeting IGFBP5 was cloned into a pRNATU6.1-Neo-cGFP plasmid (GenScript, Piscataway, NJ, USA)), and the resulting vector, pRNATU6.1-Neo-cGFP-shIGFBP5, was transduced into A2058 melanoma cells with Lipofectamine^®^ 2000. The shRNA sequences targeting IGFBP5 are provided in Table S3. Control and scramble G418-resistant clones of A375 and A2058 cells were generated by transfection with empty pcDNA3.1-Neo-EGFP and pRNATU6.1 vectors, respectively. The transfected cells were selected for 2 weeks with G418, and individual colonies were isolated and grown. All constructs were verified by restriction enzyme digestion and standard DNA sequencing.

### Fluorescence activated cell sorting (FACS)

Subconfluent cells were transfected with either pcDNA3.1-Neo-EGFP or pcDNA3.1-Neo-EGFP-IGFBP5 for A375 cells and either pRNATU6.1-Neo-cGFP-scramble or pRNATU6.1-Neo-cGFP-shIGFBP5 for A2058 cells. Cells were harvested and sorted according to GFP fluorescence with a flow cytometer (BD Biosciences, Franklin Lakes, NJ, USA). Additionally, the stem cell surface marker CD133 was used for cell sorting with the anti-CD133-APC antibody (130-098-829, Miltenyi Biotec, Bergisch Gladbach, Germany).

### Western blots

Western blots were performed as previously described [54]. The following antibodies were used: IGFBP5 (ab4255, Abcam, Cambridge, UK), vimentin (ab8069, Abcam), GAPDH (ab75834, Abcam), HIF1α (NB100-134, Novus, St. Louis, MO, USA), IGF1R-P(Try1135/1136)(3024, Cell Signaling Technology (CST), Danvers, MA, USA), ERK1/2-P (Thr202/Tyr204) (4376, CST), ERK1/2 (4695, CST), E-cadherin (3195, CST), p38-MAPK-P (Thr180/Tyr182) (9215, CST), p38-MAPK (9212, CST), sheep anti-mouse IgG (ZB-2305, ZSGB-Bio, Beijing, China), and sheep anti-rabbit IgG (ZB-2301, ZSGB-Bio). Enhanced chemiluminescence substrate kit (RPN2232, GE Healthcare, Piscataway, NJ, USA) was used for the chemiluminescent detection of signals with BioMax film (Kodak, Rochester, NY, USA).

### Immunofluorescence

Immunofluorescence was performed as previously described [55]. Briefly, cells were incubated for 1 h at room temperature with the primary antibodies IGFBP5, E-cadherin, and vimentin. Next, the cells were washed three times with PBS followed by incubation with Alexa Fluor^®^ 594 goat anti-rabbit IgG (A-11037, Life Technologies) for 1 h. After washing with PBS, the cells were examined using a Living Cell Imaging System (UltraVIEW VoX, Perkin-Elmer, Waltham, MA, USA). Cell nuclei were visualized using 2 μM 4, 6-diamidino-2-phenylindole (DAPI) (Sigma-Aldrich, St. Louis, MO, USA).

### Immunohistochemistry and H&E staining

Immunohistochemical staining was used to assess protein expression levels in the tissues. Formalin-fixed paraffin-embedded tissue sections collected from PLA General Hospital were de-paraffinized in xylene, rehydrated through graded ethanol, and boiled for 10 min in citrate buffer (10 mM, pH 6.0) for antigen retrieval. Endogenous peroxidase activity was suppressed by exposure to 3% hydrogen peroxide for 10 min. The slides were then blocked with 5% BSA, incubated with diluted IGFBP5 polyclonal primary antibody for 1 h at 37°C and finally incubated with goat-anti-rabbit IgG (PV-9000, ZSGB-Bio, Beijing, China) for 20 min. The tissue was visualized with 3, 3′-diaminobenzidine (DAB) stain and counter-stained with H&E for microscopic examination. Immunohistochemical staining and scoring of the staining intensity was performed as previously described and blinded to any clinical data [18]. Briefly, staining score was rated as 0 (no staining), 1 + (weak), 2 + (moderate) or 3 + (strong).

### Transwell migration and invasion assays

A375 cells transfected with pcDNA3-Neo-EGFP-IGRBP5 or empty pcDNA3-Neo-EGFP vector were plated at 1 × 10^5^ per well into the upper transwell chambers and 20% FBS-containing medium was placed into the bottom chamber. After incubation at 37°C in 5% CO_2_ for 12 h, the cells remaining at the upper surface of the membrane were removed with a cotton swab. The cells that migrated through the 8-mm sized pores and adhered to the lower surface of the membrane were fixed with 4% paraformaldehyde, stained with H&E, and photographed. Transwell invasion assays were conducted as indicated for the migration assay, with the exception that the upper chamber was coated with Matrigel (dilutions range from 1:5 - 1:8, BD Biosciences) in the invasion assay.

### Colony formation assays and cell counting kit-8 (CCK-8) assays

Cells were harvested seeded at 500 cells/well in 6-well plates and incubated at 37°C in a 5% CO_2_, humidified incubator for 12 days. The medium was changed in 3-day intervals. At the end of the incubation period, the cultures were fixed with 4% paraformaldehyde and stained with crystal violet. Cell viability was measured for A375 and A2058 cells, both control and those with stable IGFBP5 expression, using the Cell Counting Kit-8 from Dojindo (CK04, Kumamoto, Japan) according to the manufacturer's instructions. After treatment with CCK-8, the absorbance was measured at 490 nm with a microplate reader. The absorbance values from the vector control cells were used as references.

### Xenograft models

For tumorigenicity detection, 12 female SCID/beige mice (4-week old, Beijing Laboratory Animal Center, Beijing, China), were randomly divided into two groups (6 mice/group). Both groups received subcutaneous injections of either pcDNA3.1-Neo-EGFP vector control or A375 IGFBP5 OE cells (1 × 10^7^ cells in 200 μL PBS) and shRNA vector or shIGFBP5 A2058 cells (5 × 10^6^ cells in 200 μL PBS), respectively. Tumor growth was evaluated by measuring the length and width of the tumor mass with calipers every 3 days. After approximately 30 days, the mice were sacrificed, and tumor weights were evaluated with an analytical balance. All of the animal experiments were approved by the Institutional Review Board of the Beijing Institute of Genomics, CAS, in accordance with the National Institutes of Health (NIH) Guide for the Care and Use of Laboratory Animals.

### Tumor metastasis assays (*in vivo*)

The empty vector control or A375 IGFBP5 OE A375 cells and shRNA vector or shIGFBP5 A2058 cells were injected into the caudal veins of mice (5 × 10^6^ cells in 200 μL PBS, 6 mice/group). SCID mice were inspected every 3 days, killed after 6-7 weeks, and their lungs were dissected, fixed with 10% buffered formalin, and prepared for histological analysis. All experimental procedures involving animals were in accordance with the Guide for the Care and Use of Laboratory Animals (NIH publication no. 80–23, revised 1996).

### Transcriptome-sequencing and bioinformatics analysis

Whole-transcriptome sequencing was conducted using Ion Proton™ sequencing system (Life Technologies). All cDNA libraries were constructed using the Ion Total RNA-Seq Kit v2 (4479789, Life Technologies) protocol; sequencing templates were prepared using the Ion One Touch™ 2 system and the sequencing procedure was conducted according to the Ion PI™ chip manufacturer's instructions. We used Cutadapt (v1.4.2) to ensure clean data and remove any reads shorter than 35 bp or with quality scores of less than 17. Raw reads were mapped to the reference human genome using TopHat (v2.0.13) and Bowtie 2 (v2.2.4), reaching an average mapping rate of 96%. Total mapped reads were normalized using Cufflinks (v2.2.0) and SAMtools (v1.1), followed by calculation of differentially expressed reads with Cuffdiff set with the filters: *P*-value ≤ 0.05 and fold change ≥ 2. The resulting UniGenes were further annotated using the Gene Ontology (GO) database and Ingenuity Pathway Analysis (IPA, http://www.ingenuity.com/). The normalized RNA expression data for IGFBP5 overexpression in A375 cells were deposited in the Gene Expression Omnibus (GEO) database (accession number, GSE64693).

### Statistical analysis

The two-tailed Student's t-test was used to analyze the experimental data, including qRT-PCR assay, cell proliferation, transwell assay, FACS assay and xenograft model analysis. The two sample Wilcoxon Rank-Sum test (Mann–Whitney) was used to compare the immunohistochemical scores for all pigment nevus samples and melanoma samples. Data were presented as the mean ± standard deviation (SD). *p* < 0.05 was considered statistically significant.

## SUPPLEMENTARY FIGURES AND TABLES


