# Supplementary material for: Insulin-like growth factor binding protein 5 (IGFBP5) functions as a tumor suppressor in human melanoma cells
Source: Oncotarget. 2015 May 12;6(24):20636–49. doi: 10.18632/oncotarget.4114 (PMC4653031; doi:10.18632/oncotarget.4114)
Supplement: Supplementary file 1 [file oncotarget-06-20636-s001.pdf]

Insulin-like growth factor binding protein 5 (IGFBP5) functions as a tumor suppressor in human melanoma cells

Supplementary Material

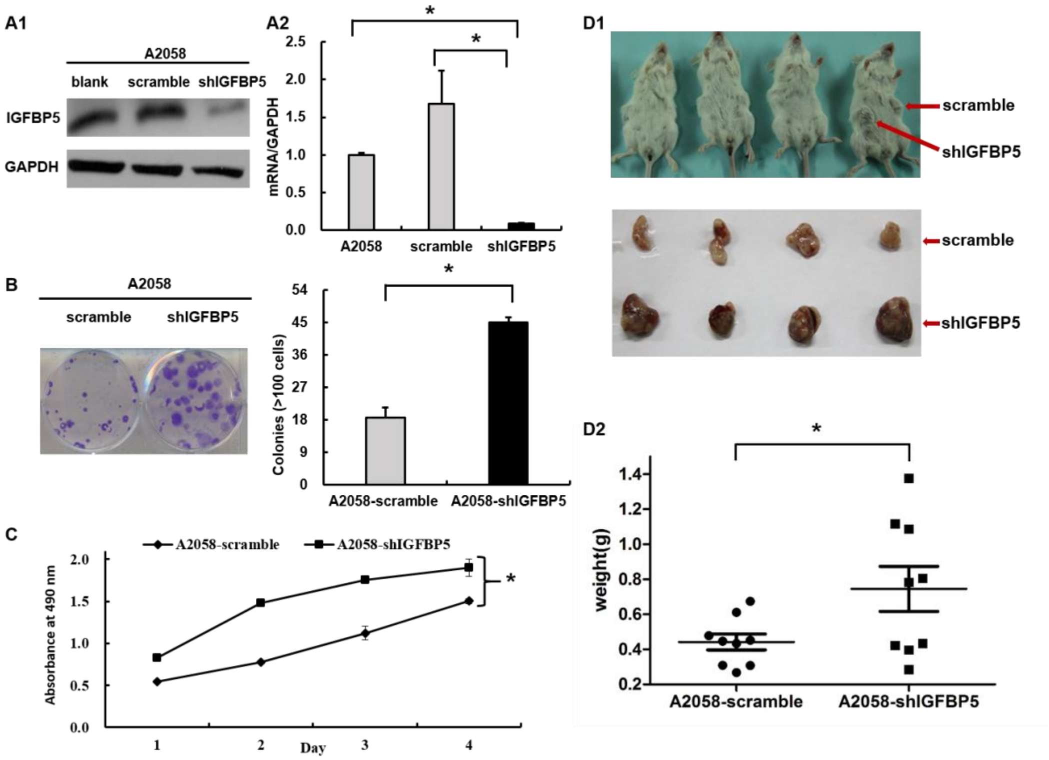

Supplementary Figure S1: Knockdown of IGFBP5 in A2058 cells promotes cell proliferation both *in vitro* and *in vivo.*

(A1 and A2) A2058 cells were transfected with IGFBP5-silencing shRNA sequences and non-functional scramble control. Western blots and qRT-PCR analysis detecting IGFBP5 expression in cells transfected with control vectors and shRNA sequences were shown. Colony formation assay (B), CCK-8 assay (C) were used to explore the effects of stable IGFBP5 knockdown on melanoma cell proliferation *in vitro*. (D1 and D2) Xenograft assay was conducted to explore the effects of IGFBP5 knockdown on A2058 cell proliferation *in vivo*. Down-regulation of IGFBP5 increased the tumor growth of melanoma A2058 cells significantly. Data were shown as mean ± SD from three independent experiments. *P*-values based on two-side Student *t*-test comparing control and IGFBP5 knockdown. *, $P < 0.05$.

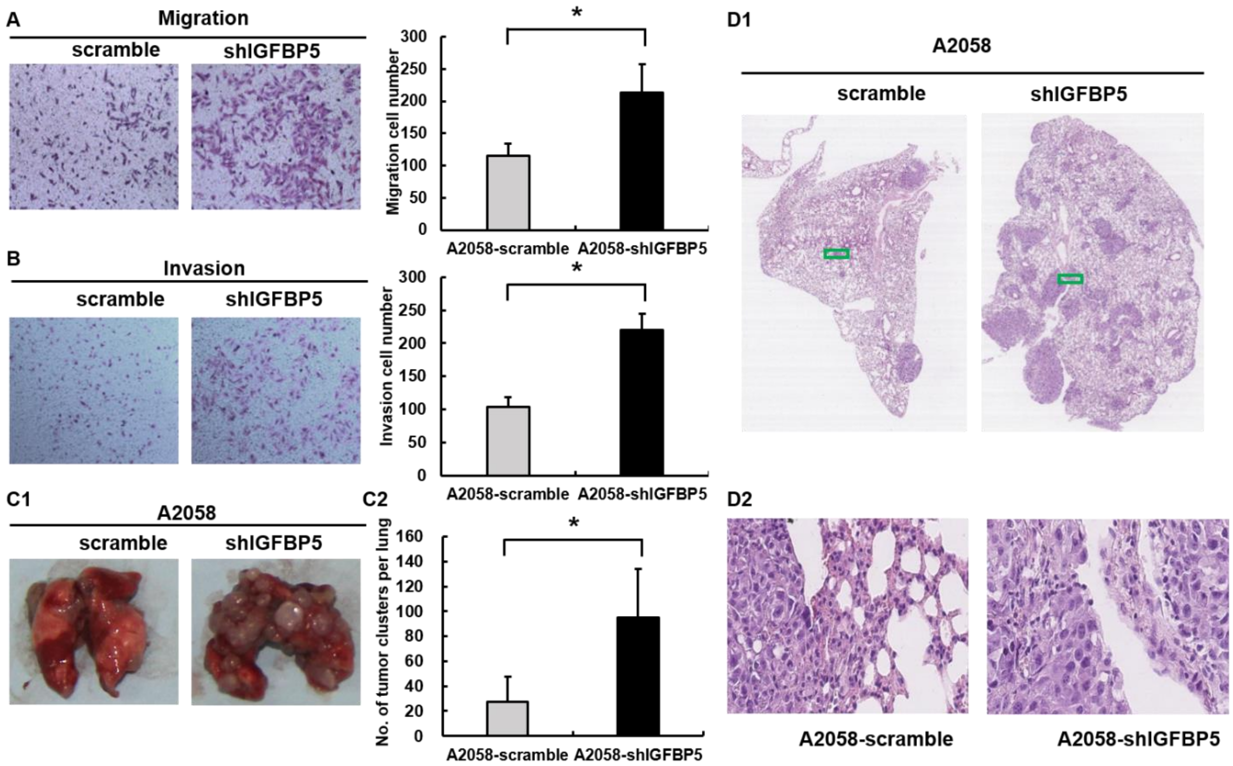

Supplementary Figure S2: Down-regulation of IGFBP5 enhances cell migration and invasion *in vitro* and promoted pulmonary metastasis *in vivo* in A2058 cells.

(A and B) Transwell cell migration assay and Matrigel cell invasion assay were conducted by using stable IGFBP5 knockdown cells and control cells. Representative images of cells stained with H&E (left) and the mean of migrated or invaded cells (right) were shown. Data were shown as mean ± SD from three independent experiments. *, $P < 0.05$. (C1) Representative images of the lungs harvested from the melanoma cell-injected mice were shown. (C2) The mean number of metastatic lung clusters from mice control and A2058 IGFBP5 KD tumors were plotted, as analyzed by H&E staining. Data were shown as mean ± SD from repeated experiments. *, $P < 0.05$. (D) Representative H&E staining of lung tumor sections and adjacent tissues from IGFBP5 knockdown mice and control mice. The images in D2 were the magnifications of the two green boxes in D1.

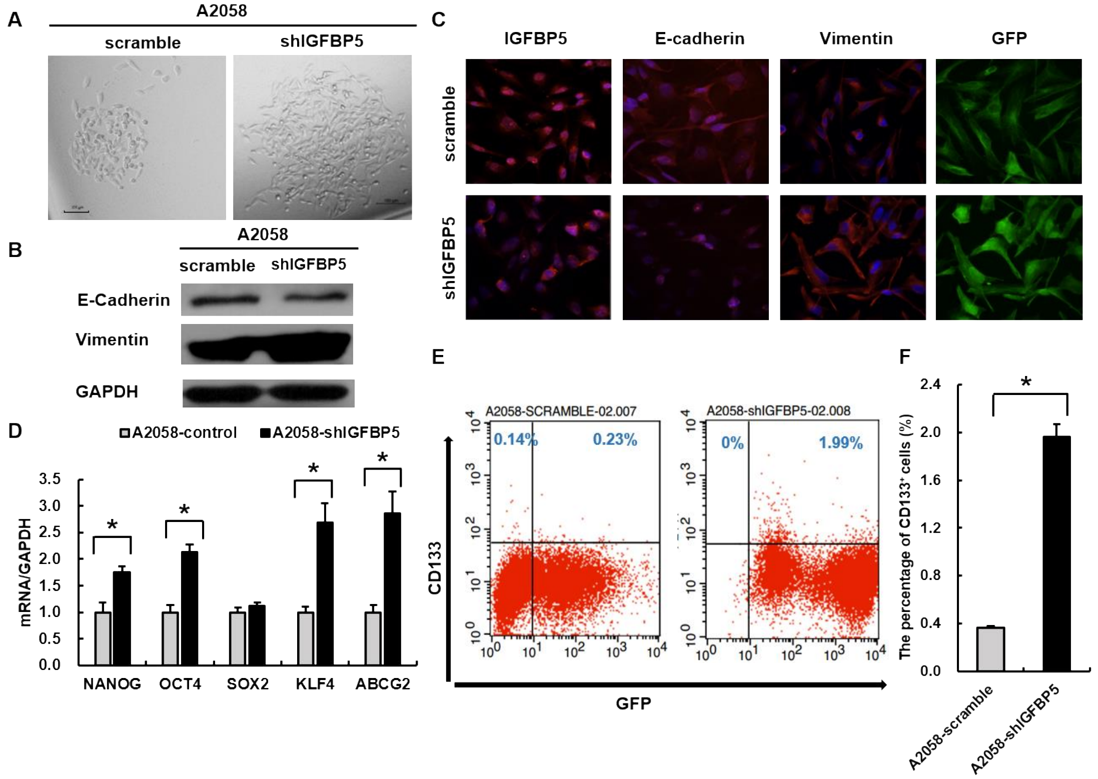

Supplementary Figure S3: Knockdown of IGFBP5 promotes EMT and stem cell features of tumor cells.

(A) The characteristic morphologies of A2058 control and IGFBP5 knockdown cell clones were shown. Down-regulation of IGFBP5 in A2058 cells resulted in an elongated fibroblast-like morphology. Magnification, × 200. (B, C) Expression of EMT markers analyzed by western blots and immunofluorescence in A2058 control cells and IGFBP5 knockdown cells. Nuclei were shown with DAPI staining. Magnification, × 400. (D) Up-regulation of the representative stem cell markers *NANOG*, *OCT4*, *KLF4*, and *ABCG2* in A2058 IGFBP5 knockdown cells via qRT-PCR analysis. Data were shown for the mean ± SD from three independent experiments. *P* values based on two-side Student *t*-test comparing A2058 IGFBP5 knockdown cells and control cells. *, $P < 0.05$. (E) FACS analysis of the stem cell marker CD133 in A2058 IGFBP5 knockdown cells and control cells. (F) Graph demonstrates the mean ± SD for the percent of $CD133^+$ cells in A2058 IGFBP5 KD cells from three independent experiments. *, $P < 0.05$.

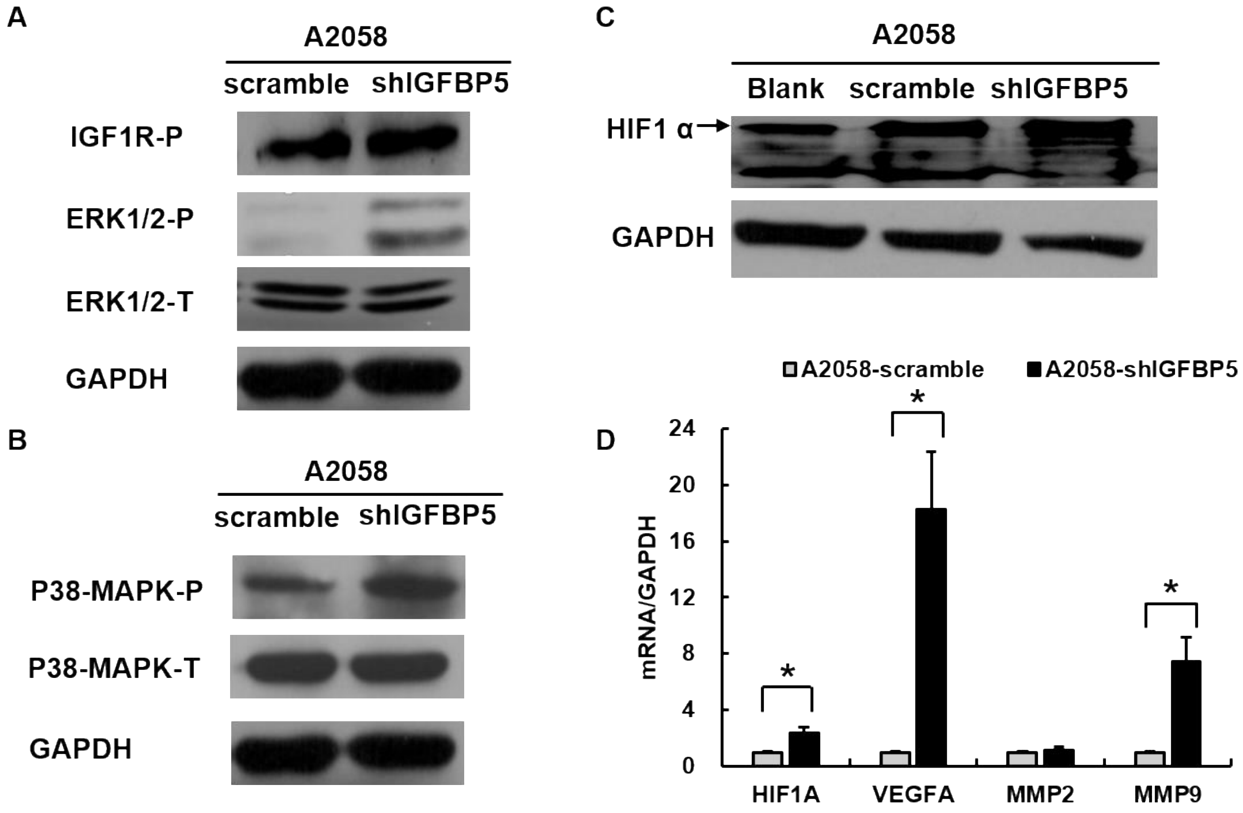

Supplementary Figure S4: Down-regulation of IGFBP5 promotes HIF1α expression via p38-MAPK and ERK signaling pathways.

(A and B) Western blots assay for the phosphorylation state of IGF1R, ERK1/2, and p38-MAPK in A2058 IGFBP5 knockdown cells. Phosphorylation of IGF1R, ERK1/2 and p38-MAPK was increased in A2058 IGFBP5 knockdown cells. (C) Knockdown of IGFBP5 increased expression of HIF1α visualized by western blots. The arrow points to the band of HIF1α. (D) qRT-PCR analysis of target genes of HIF1α, *VEGFA* and *MMP9*, in A2058 IGFBP5 knockdown cells. Data were shown as mean ± SD from three independent experiments. *, $P < 0.05$

Supplemental Table S1: The list of top 5 biological functions relevant to IGFBP5 overexpression in A375 cells by IPA analysis

**Molecular and Cellular Functions**	***P*-value**	**No. of Molecules**
Cellular Movement	2.11E-32 - 3.44E-06	177
Cellular Growth and Proliferation	4.11E-20 - 3.10E-06	221
Cell Death and Survival	4.71E-20 - 2.98E-06	202
Cellular Development	3.07E-18 - 3.16E-06	229
Cell-To-Cell Signaling and Interaction	3.52E-14 - 3.37E-06	129

Supplementary Table S2: Primer sequences for quantitative real-time PCR.

Gene	Strand	Primer sequences
HIF1A	Forward	5'-TCCATGTGACCATGAGGAAA-3'
	Reverse	5'-CCAAGCAGGTCATAGGTGGT-3'
IGFBP5	Forward	5'-TGACCGCAAAGGATTCTACAAG-3'
	Reverse	5'-CGTCAACGTACTCCATGCCT-3'
NANOG	Forward	5'-ACAACTGGCCGAAGAATAGCA-3'
	Reverse	5'-GGTTCCCAGTCGGGTTCAC-3'
OCT4	Forward	5'-GGGTTTTTGGGATTAAGTTCTTCA-3'
	Reverse	5'-GCCCCCACCCTTTGTGTT-3'
SOX2	Forward	5'-CCCACCTACAGCATGTCCTACTC-3'
	Reverse	5'-TGGAGTGGGAGGAAGAGGTAAC-3'
KLF4	Forward	5'-GAAATTCGCCCGCTCCGATGA-3'
	Reverse	5'-CTGTGTGTTTGCGGTAGTGCC-3'
ABCG2	Forward	5'-CAGTGTCACAAGGAAACACC-3'
	Reverse	5'-GAGACCAGGTTTCATGATCC-3'
FOXA1	Forward	5'-GAAGATGGAAGGGCATGAAA-3'
	Reverse	5'-GCCTGAGTTCATGTTGCTGA-3'
VEGFA	Forward	5'-AGGGCAGAATCATCACGAAGT-3'
	Reverse	5'-AGGGTCTCGATTGGATGGCA-3'
MMP2	Forward	5'-TCTCCTGACATTGACCTTGGC-3'
	Reverse	5'-CAAGGTGCTGGCTGAGTAGATC-3'
MMP9	Forward	5'-GGGACGCAGACATCGTCATC-3'
	Reverse	5'-TCGTCATCGTCGAAATGGGC-3'
GAPDH	Forward	5'-AATGAAGGGGTCATTGATGG-3'

	Reverse	5'-AAGGTGAAGGTCGGAGTCAA-3'

HIF1A, hypoxia-inducible factor 1-alpha ; IGFBP5, insulin-like growth factor binding protein 5; NANOG, nanog homeobox; OCT4, octamer-binding transcription factor 4; SOX2, SRY (sex determining region Y)-box 2; KLF4, kruppel-like factor 4; ABCG2, ATP-binding cassette sub-family G member 2; FOXA1, forkhead box protein A1; VEGFA, vascular endothelial growth factor A; MMP2, matrix metalloproteinase 2; MMP-9, matrix metalloproteinase 9; GAPDH, glyceraldehyde 3-phosphate dehydrogenase.

Supplementary Table S3: Primer designs of shRNA sequences for targeting IGFBP5.

Name	Strand	Primer sequences
Scramble	Sense	5’-GATCCGCACTACCAGAGCTAACTCAGATAGTACTTCAAGAGAGTACTATCTGAGTTAGCTCTGGTAGTGCTTTTTA-3’
	Antisense	5’-AGCTTAAAAAGCACTACCAGAGCTAACTCAGATAGTACTCTCTTGAAGTACTATCTGAGTTAGCTCTGGTAGTGCG-3’
shRNA-1	Sense	5’-GATCCCTGTGTACCTGCCCAATTGTTCAAGAGACAATTGGGCAGGTACACAGTTTTTA-3’
	Antisense	5’-AGCTTAAAAACTGTGTACCTGCCCAATTGTCTCTTGAACAATTGGGCAGGTACACAGG-3’
shRNA-2	Sense	5’-GATCCGAAGCTGACCCAGTCCAAGTCAAGAGCTTGGACTGGGTCAGCTTCTTTTTA-3’
	Antisense	5’-AGCTTAAAAAGAAGCTGACCCAGTCCAAGCTCTTGACTTGGACTGGGTCAGCTTCG-3’

shRNA, short hairpin RNA.
